# Irradiation-induced telomerase activity and gastric cancer risk: a case-control analysis in a Chinese Han population

**DOI:** 10.1186/1471-2407-10-312

**Published:** 2010-06-21

**Authors:** Xianli He, Qing Qiao, Naijian Ge, Jing Nan, Shuqun Shen, Zizhong Wang, Yefa Yang, Guoqiang Bao

**Affiliations:** 1 Department of General Surgery, Tangdu Hospital, The Fourth Military Medical University, Xi'an 710038, China; 2 Department of Interventional Radiology 1, Eastern Hepatobiliary Surgery Hospital, The Second Military Medical University, Shanghai 200438, China; 3 Department of Epidemiology, The Fourth Military Medical University, Xi'an 710032, China

## Abstract

**Background:**

Telomerase expression is one of the characteristics of gastric cancer (GC) cells and telomerase activity is frequently up-regulated by a variety of mechanisms during GC development. Therefore, we hypothesized that elevated levels of activated telomerase might enhance GC risk due to increased propagation of cells with DNA damage, such as induced by γ-radiation.

**Methods:**

To explore this hypothesis, 246 GC cases and 246 matched controls were recruited in our case-control study. TRAP-ELISA was used to assess the levels of telomerase activity at baseline and after γ-radiation and the γ-radiation-induced telomerase activity (defined as after γ-irradiation/baseline) in cultured peripheral blood lymphocytes (PBLs).

**Results:**

Our data showed that there was no significant difference for the baseline telomerase activity between GC cases and controls (10.17 ± 7.21 *vs. *11.02 ± 8.03, *p *= 0.168). However, after γ-radiation treatment, γ-radiation-induced telomerase activity was significantly higher in the cases than in the controls (1.51 ± 0.93 *vs*. 1.22 ± 0.66, *p *< 0.001). Using the median value of γ-radiation-induced telomerase activity in the controls as a cutoff point, we observed that high γ-radiation-induced telomerase activity was associated with a significantly increased GC risk (adjusted odds ratio, 2.45; 95% confidence interval, 1.83-3.18). Moreover, a dose response association was noted between γ-radiation-induced telomerase activity and GC risk. Age, but not sex, smoking and drinking status seem to have a modulating effect on the γ-radiation-induced telomerase activities in both cases and controls.

**Conclusion:**

Overall, our findings for the first time suggest that the increased γ-radiation-induced telomerase activity in PBLs might be associated with elevated GC risk. Further confirmation of this association using a prospective study design is warranted.

## Background

Telomerase is a specialized ribonucleoprotein consisting of a RNA subunit (telomerase RNA [TR]) and a rate-limiting catalytic protein subunit (telomerase reverse transcriptase [TERT])[1]. A well-established function of telomerase is to synthesize telomeric repeats onto chromosomal ends and thus be responsible for the telomere maintenance [2]. In most normal human somatic cells, telomerase activity is not detectable or at very low level[3]; however, telomerase activity has been confirmed in approximately 85% of patients suffering from the most common cancers, such as breast, prostate, lung, liver, colon and gastric cancers[4]. The increased telomerase activity in cancer cell has been showed to help maintain the telomere length of cancer cell at a constant length over indefinite cell division. Moreover, several laboratories demonstrated that the over-expression of telomerase induced tumor formation in both mouse and human models[5,6]. Such above observations provide strong supports that the activation of the telomerase is a critical event in human cell immortalization and carcinogenesis.

Besides maintaining telomere length, several recent reports indeed suggested that the activation of telomerase served other important functions in multi-step process of tumor development[7]. It has been reported that telomerase may promote cell proliferation by modulating expression of growth-controlling genes, such as EGFR, FGF and IL-1Ra [8]. This might correspond with observations from numerous groups that telomerase activity correlates with the stage and grade of many human malignancies[9,10]. In addition, telomerase expression has also been linked to chromosome healing and increased cell proliferation and survival[11,12]. Beyond a link to proliferation, high telomerase activity reflects a deregulation of cell cycle associated with an increased rate of cells entering S phase and a higher degree of malignancy[13]. More importantly, telomerase expression could be induced by some environmental exposures, such as irradiation and N-nitrosobis(2-oxopropyl)amine[14]. Therefore, we hypothesized that cells with increased telomerase activity might be able to undergo more replications and thereby accumulate more mutations so that individuals with enhanced induction of telomerase activity might be at higher risk for the development of cancer. In the present study, we used γ-radiation, which can cause oxidative damage and induce single- or double-strand breaks, to mimic environmental mutagen for the treatment of PBLs. Actually, the level of γ-radiation-induced telomerase activity is only a biomarker for the evaluation of the inherited inducibility of telomerase activity, but not truly clinically relevent increase.

Gastric cancer is one of the most popular malignant tumors in China. Although more and more environmental risk factors[15], such as cigarette smoking, alcohol consumption, *Helicobacter pylori *(Hp) infection and excessive salt intake, have been identified, the genetic factors associated with sporadic gastric cancer remains mostly unclear. In this study, we designed a case-control study to investigate whether inherited discrepancy on γ-radiation-induced telomerase activity in normal cells is associated with GC risk.

## Methods

### Study population

This analysis included 246 GC patients and 246 healthy controls. The cases who were newly diagnosed with histologically confirmed primary GC were consecutively recruited from Tangdu hospitals affiliated to The Fourth Military Medical University, Xi'an, Shaanxi, China, between June 2007 and October 2008. None of the cases underwent prior chemotherapy or radiotherapy. No age, sex, or disease stage restrictions for case recruitment were used. Two hundred and nineteen patients (89%) had adenocarcinoma and 27 patients (11%) had other histological types, including squamous cell carcinoma and undifferentiated carcinoma etc. Twenty-five patients (10%) had stageⅠdisease, 54 patients (22%) had stageⅡdisease, 103 patients (42%) had stage Ⅲ disease and 64 patients (26%) had stage Ⅳ disease. Healthy controls with no previous cancer history were enrolled during the same time period as cases recruited from individuals who visited the same hospital for physical examination with a response rate of about 75%. The controls were frequency-matched to the cases on age (±5 years), sex, and the residential areas. Ethnicity of all participants was Chinese Han. After signed informed consent was obtained from each individual, all participants were interviewed to collect information on demographics, smoking history and alcohol consumption using standard questionnaire. After interview, venous blood sample from each subject was drawn into coded tubes (5 mL into heparinized tube and 2 mL into regular tube) and delivered to the laboratory for analysis. Researchers doing the laboratory assays were blinded to the case-control status of the samples. This research was approved by the institutional review board of The Fourth Military Medical University.

### Measurement of serum antibody IgG to H. pylori

The 2 mL of coagulated blood was centrifuged for 10 min at 400 × g to collect the serum. The serum was then divided into three aliquots for storage in -80°C. Hp infection in all subjects was determined by *pylori *DTect test using a commercial IgG enzyme-linked immunosorbent assay kits (Diagnostic Technology, Pymble, Australia) according to the manufacturer's instruction. The test has been validated in Chinese populations with a high sensitivity and specificity for detection of *H. pylori *infection[16]. In China, most of patients with *H.pylori* infection do not get any treatment except for the patients with gastric ulcer. Therefore, in our study, we did not collect the information on the treatment of *H.pylori* infection in all subjects. We do believe that the influence of *H.pylori* treatment on the findings of our study should be very limited due to the small percentage.

### Lymphocyte isolation and cell culture

In our study, lymphocyte was chosen as a surrogate tissue to evaluate the inherited inducibility of telomerase activity that could be mainly affected by individual's genetic variation, but not either tumor cells, which could not represent normal genetic background, or normal gastric mucosa that is not easy to obtain for analysis. Lymphocytes were isolated from the 5 mL of whole blood (anticogulated) using standard Ficoll-Hypaque techniques and then stored in liquid nitrogen at 4 × 10^6 ^cells per vial. The lymphocytes were cultured as previously described [17] with a minor modification. In brief, the thawed lymphocytes were incubated in RPMI 1640 supplemented with 20% fetal bovine serum and 100 μg/mL phytohemagglutinin (PHA) (Sigma) at 37°C for 96 hours. For each sample, 4 × 10^6 ^lymphocytes were equally cultured in two flasks. Cultured lymphocytes were irradiated through direct exposure to γ-radiation using a ^60^Co source at an optimal dose of 0.5 Gy and then allowed to grow for an extra 12 hours before being harvested. Unirradiated lymphocytes were also harvested at the same time. The total protein was extracted from cultured lymphocytes and the protein concentration was determined using the BCA Protein Assay (Thermo Fisher Scientific Inc., Rockford, IL).

### Determination of telomerase activity

Telomerase activity was determined using the telomerase TRAP-ELISA^plus ^kit (Boehringer Mannheim) according to the manufacturer's instructions. In comparision with recently developed flourescent real-time PCR-based assay, TRAP-ELISA assay exhibited a stable and controllable reproducibility[18]. In brief, the equal amount (0.4 μg) of protein from each sample was incubated with a biotinylated telomerase substrate oligonucleotide (P1-TS primer) at 25°C for 20 minutes. At the same time, a heat-treated (85°C for 10 minutes) negative control was included for each sample during incubation. Then, the extended products were amplified using polymerase chain reaction (PCR) with P1-TS and P2 primers. The PCR conditions were 30 cycles of 94°C for 30 seconds, 60°C for 30 seconds, and 72°C for 90 seconds performed on a TC-96 thermocycler (Bioer technology Co., Hangzhou, China). After 12 minutes of denaturation, the PCR-amplified products for each sample were separately hybridized with buffer T and buffer IS at 37°C for 2 hours and immobilized onto streptavidin-coated microtiter plates; the negative controls were only hybridized with buffer T. After this step, all of the wells on the plates were incubated with a peroxidase-labeled anti-digoxigenin polyclonal antibody at room temperature for 30 minutes. Finally, the absorbance of each well was measured at a wavelength of 450 nm (reference wavelength, 595 nm) after the addition of a peroxidase substrate (3,3',5,5'-tetramethylbenzidine). For each plate, one positive control was set for a calibrator in order to standardize between different runs. The relative telomerase activity within each sample was calculated as follows: (the absorbance of the sample - the absorbance of the heat-treated sample)/the absorbance of the internal standard of the sample.

### Statistical analysis

All statistical analyses were done using the Statistical Analysis System (SAS) (Version 9.1.3; SAS Institute Inc. Cary, NC). Smoking and drinking status were categorized as dichotomized variables. Individuals who had smoked less than 100 cigarettes in his or her lifetime were defined as never smokers, and those that consumed 3 and more standard cups a week for over 6 months were considered as ever drinkers. We evaluated the difference between the cases and controls in the distribution of categorical variables (sex, Hp antibody positivity, smoking and drinking status) and continual variables (age, pack-years and telomerase activity) using the Pearson χ2 test and the Student *t*-test, respectively. The γ-radiation-induced telomerase activity (defined as the value after γ-radiation/baseline value) was also analyzed as a categorical variable by grouping it based on the median or tertile values in the controls. The association between GC risk and γ-radiation-induced telomerase activity was estimated using odds ratios (ORs) along with corresponding 95% confidential intervals (CIs). To adjust for the confounding effects of age, sex, Hp antibody positivity, smoking and drinking status, unconditional logistic regression analysis with multiple covariates was performed. Stratified analyses were performed to compare γ-radiation-induced telomerase activity among different subgroups of cases or controls, to assess the GC risk associated with γ-radiation-induced telomerase activity in those subgroups. The a-priori statistical power was calculated to determine the magnitude of difference in telomerase activity which could be reasonably detected. Given the sample size of the current study, we anticipated 96% power to detect a 20% change in γ-radiation-induced telomerase activity between GC cases and controls at the 0.05 significance level. A *p*-value of less than 0.05 (two-sided) was considered statistically significant.

## Results

Selected characteristics of the 246 GC patients and 246 healthy controls are shown in Table 1. The cases and controls were well matched with respect to sex (*p *= 1.000) and age (*p *= 0.375). However, there were statistically significant differences between the cases and the controls in terms of smoking status, drinking status, Hp antibody positivity and pack-years. More patients with GC than controls reported having a history of smoking (55% *vs*. 41%; *p *= 0.002) and drinking (51% *vs*. 40%; *p *= 0.019). GC patients had a significantly higher percentage of Hp antibody positivity than controls (68% *vs*. 52%; *p *< 0.001). In addition, GC patients were self-reported heavier smokers than control subjects (42.8 ± 27.3 *vs*. 27.1 ± 16.5, *p *< 0.001).

**Table 1:** Selected characteristics of GC cases and controls

Variables	cases (*n *= 246)	controls (*n *= 246)	***p ***^a^
Sex, *n *(%)			
Male	175 (71)	175 (71)	
Female	71 (29)	71 (29)	1.000
Smoking status, *n *(%)			
Never	110 (45)	145 (59)	
Ever	136 (55)	101 (41)	0.002
Drinking status, *n *(%)			
Never	121 (49)	147 (60)	
Ever	125 (51)	99 (40)	0.019
Hp antibody positivity, *n *(%)			
Yes	167 (68)	128 (52)	
No	79 (32)	118 (48)	<0.001
Mean age, years (SD)	60.1 (11.2)	59.8 (10.8)	0.375
Mean pack-years (SD)^c^	42.8 (27.3)	27.1(16.5)	<0.001

SD: standard deviation.

^a ^*p *values were derived from the Pearson χ2 test for categorical variables (sex, Hp antibody positivity, smoking status and drinking status ) and Student's *t *test for continuous variables (age and cigarette smoking pack-years).

^b ^Ever smokers only.

We used the telomerase PCR ELISA^plus ^kit to measure the telomerase activity at baseline and after γ-radiation and the γ-radiation-induced telomerase activity in PBLs (Table 2). We found that there is no significant difference for the mean baseline telomerase activity in PBLs between cases and controls (10.17 ± 7.21 *vs*. 11.02 ± 8.03; *p *= 0.168). However, after γ-radiation treatment, the telomerase activity was significantly higher in the GC cases than in the controls (*p *< 0.001). Similar results for the γ-radiation-induced telomerase activity (after γ-radiation/baseline) was observed in the GC cases and controls, indicating that γ-radiation-induced telomerase activity was significantly higher in the GC cases than in the controls (1.51 ± 0.93 *vs*. 1.22 ± 0.66; *p *< 0.001). In addition, our data showed that the after-γ-radiation telomerase activity increased significantly in both GC cases (15.36 ± 12.05 *vs*. 10.17 ± 7.21; *p *< 0.001) and healthy controls (13.44 ± 10.22 *vs*. 11.02 ± 8.03; *p *< 0.001) when compared with the baseline levels.

**Table 2:** Telomerase activity in cases and controls

Telomerase activity	GC cases			Controls			*p*^b^
		
	*n*	Mean (SD)	*p*^a^	*n*	Mean (SD)	*p*^a^	
Baseline	246	10.17 (7.21)		246	11.02 (8.03)		0.168
After γ-radiation	246	15.36 (12.05)	<0.001	246	13.44 (10.22)	<0.001	<0.001
γ-Radiation-induced	246	1.51 (0.93)		246	1.22 (0.66)		<0.001

SD: standard deviation.

^a^*p *values were calculated for assessing the difference in telomerase activity between baseline and after γ-radiation in

GC cases and controls.

^b^*p *values were derived from the student *t*-test for the difference between the GC cases and controls.

Furthermore, we performed unconditional logistic regression analysis to assess the association between the γ-radiation-induced telomerase activity and GC risk (Table 3). We first dichotomized the γ-radiation-induced telomerase activity into high and low groups by arbitrarily using the median value in the controls as the cutoff. After the adjustment for the confounding effects of age, sex, Hp antibody positivity, smoking status, and drinking status, we found that individuals with high γ-radiation-induced telomerase activity had a significantly increased risk of GC, with an OR of 2.45 (95% CI, 1.83-3.18). Next, we arbitrarily categorized the subjects into three groups based on the tertile values of γ-radiation-induced telomerase activity in the controls. We observed a dose-response relationship between GC risk and γ-radiation-induced telomerase activity. The chi-square tests for trend were significant (*p *< 0.001). Using the subjects in lowest tertile as reference, the adjusted ORs (95% CIs) for individuals in middle and highest tertiles of γ-radiation-induced telomerase activity were 1.94 (95% CI, 1.55-2.46) and 3.87 (95% CI, 2.41-6.15), respectively.

**Table 3:** Relative GC risk estimates for γ-radiation-induced telomerase activity

γ-radiation-induced telomerase activity	GC cases, n (%)	Controls, n (%)	OR (95% CI)^a^
By median (50th percentile)			
Low	71 (29)	123 (50)	Reference
High	175 (71)	123(50)	2.45 (1.83-3.18)
By tertile			
First tertile	36 (15)	82 (33.3)	Reference
Second tertile	71 (29)	82 (33.3)	1.94 (1.55-2.46)
Third tertile	139 (56)	82 (33.3)	3.87 (2.41- 6.15)
*P *for trend			<0.001

OR, odds ratio; CI, confidence interval.

^a ^Adjusted by age, gender, Hp antibody positivity, smoking status and drinking status.

We also assessed the γ-radiation-induced telomerase activity according to host characteristics (Table 4). A modulating effect of age on γ-radiation-induced telomerase activity was found in both cases and controls. Individuals at least 60 years old had a significantly higher mean level of γ-radiation-induced telomerase activity than did those younger than 60 years among the GC cases (1.61 ± 1.08 *vs*. 1.39 ± 0.75; *p *= 0.060) and healthy controls (1.36 ± 0.76 *vs*. 1.07 ± 0.60; *p *< 0.001). There were no significant associations between γ-radiation-induced telomerase activity and sex, smoking status, drinking status and Hp antibody positivity in either GC cases or controls. In addition, our results showed that the level of γ-radiation-induced telomerase activity in cases was not significantly associated with the tumor stage and histological type, suggesting that telomerase activation might play a role at early stage of GC development (data not shown). We next performed the stratified analysis to examine GC risk associated with γ-radiation-induced telomerase activity by selected host characteristics (Table 5). The increased GC risk (OR [95%CI]) associated with higher γ-radiation-induced telomerase activity was more evident in male persons than in female persons (2.85 [1.98-4.48] *vs. *1.86 [1.01-3.58]) and also more evident in never-drinkers than in ever drinkers (3.35 [2.03-5.58] *vs. *1.75 [1.03-3.07]). There was no notable difference for GC risk (OR [95%CI]) associated with γ-radiation-induced telomerase activity between older peoples and younger peoples (2.49 [1.49-4.06] *vs. *2.41 [1.45-4.14]) or between never smokers and ever smokers (2.29 [1.37-3.87] *vs. *2.76 [1.67-4.59]).

**Table 4:** Stratified analysis of γ-radiation-induced telomerase activity by host characteristics

Variables	GC cases			Controls		
	
	*N*	Mean (SD)	*p*^*a*^	*N*	Mean (SD)	*p*^*a*^
Age in years						
<60	109	1.39 (0.75)		120	1.07 (0.60)	
≥60	137	1.61 (1.08)	0.060	126	1.36 (0.76)	<0.001
Sex						
Male	175	1.52 (0.88)		175	1.21 (0.64)	
Female	71	1.49 (0.97)	0.818	71	1.24 (0.70)	0.757
Smoking status						
Never	110	1.50 (0.85)		145	1.21 (0.61)	
Ever	136	1.52 (1.03)	0.873	101	1.23 (0.74)	0.826
Drinking status						
Never	121	1.47 (0.73)		147	1.19 (0.58)	
Ever	125	1.55 (1.13)	0.535	99	1.26 (0.81)	0.459
Hp antibody positivity						
Yes	167	1.48 (0.87)		128	1.20 (0.66)	
No	79	1.57 ( 1.06)	0.481	118	1.24 (0.77)	0.661

SD: standard deviation.

^a^*p *values were determined by Student's t test to assess the difference of γ-radiation-induced telomerase activity between two different subgroups in cases or controls.

**Table 5:** Estimates of GC risk associated with γ-radiation-induced telomerase activity stratified by selected variables

γ-Radiation-induced telomerase activity	Cases, n (%)	**Controls, n **(%)	OR (95% CI)^a^
Age			
< 60 years			
low	34 (31)	63 (53)	Reference
high	75 (69)	57 (47)	2.41 (1.45-4.14)
≥ 60 years			
low	37 (27)	60 (48)	Reference
high	100 (73)	66 (52)	2.49 (1.49-4.06)
Sex			
Male			
low	43 (25)	84 (48)	Reference
high	132 (75)	91 (52)	2.85(1.98-4.48)
Female			
low	28 (39)	39 (55)	Reference
high	43 (61)	32 (45)	1.86 (1.01-3.58)
Smoking history			
Never			
low	31 (28)	69 (49)	Reference
high	79 (72)	76 (51)	2.29 (1.37-3.87)
Ever			
low	40 (29)	54 (53)	Reference
high	96 (71)	47 (47)	2.76 (1.67-4.59)
Drinking status			
Never			
low	30 (25)	77 (52)	Reference
high	91 (75)	70 (48)	3.35 (2.03-5.58)
Ever			
low	41 (33)	46 (46)	Reference
high	84 (67)	53 (54)	1.75(1.03-3.07)

^a^Adjusted for age, sex, Hp antibody positivity, smoking and drinking status, where appropriate.

## Discussion

In our study, we evaluated the telomerase activity at baseline and after γ-radiation exposure in PBLs from GC patients and controls by using TRAP-ELISA. Our findings demonstrated that the cultured PBLs from cases exhibited significantly higher γ-radiation inducibility of telomerase activity than those from healthy controls. We also presented the evidence of an increased risk for GC associated with higher γ-radiation-induced telomerase activity.

Our findings demonstrated that telomerase activity significantly increased in both GC cases and controls after γ-radiation when compared with the baseline levels. This result is consistent with previous observations that telomerase activity is a γ-radiation-inducible function in hematopoietic cells[19]. Researchers have also observed the increased telomerase activity in human lymphoblasts [20] and tumor cell lines [21] after exposure to x-rays as well as in immortalized Chinese hamster cells[22] after ultraviolet irradiation. However, the signaling pathway of telomerase activation by radiation remains unclear. Finnon *et al. *[23] suggested that up-regulation of telomerase activity by x-rays in mouse leukemia cells was related to the transcription of telomerase-positive regulator proteins.

For the first time, our data demonstrated a significant association between γ-radiation-induced telomerase activity and GC development. These results are extremely consistent with previous reports [17,24], indicating that the levels of γ-radiation-induced telomerase activity detected in PBLs were statistically higher in both bladder and lung cancer cases than in corresponding controls and that these higher levels were significantly associated with an increased risk of both bladder and lung cancer. The underlying mechanisms of radiation-induced telomerase activity are still unknown. It has been suggested that both transcriptional activation and post-translational control of TERT might be involved in this process[25]. In addition, a possible role for p53 in radiation-induced telomerase up-regulation has recently been demonstrated in human lymphoblastoid cell lines[26]. Previous data produced from lung tumor and normal tissues also indicated that telomerase activation was associated with p53 over-expression[27].

The biological role of telomerase activation after the mutagen challenge remains unclear. More and more evidences showed that telomerase activation was a critical event in human cell immortalization and carcinogenesis. For example, Stewart *et al*. [28] reported that the telomerase could facilitate the tumorigenicity of ras-transformed, already-immortalized GM847 cells. In addition, transgenic mice expressing high levels of mTERT has been demonstrated to be more susceptible to tumors than littermate controls[5]. Evidence has also been accumulated in somatic dividing cells to show additional functions of telomerase that prevent cell death in light of induction and protective role of TERT against various toxic insults in mature tissues[12,29]. These observations have led to the hypothesis that elevated levels of activated telomerase might increase cancer risk by increasing propagation of cells with genomic damage. These cells will be able to undergo more replications and accumulate more mutations, thereby becoming a risk factor for cancer development.

A previous study [26] clearly indicated that γ-radiation-induced G2/M delay was more evident in cell lines from healthy controls than in cell lines from lung cancer patients. So the possibility of a cell cycle-dependent variation in telomerase activity must be considered in the interpretation of data related to the telomerase induction after irradiation. Several groups have reported that telomerase activity has no evident changes during the cell cycle[19,22,30]. Therefore, radiation-induced alterations of the cell cycle distribution can be excluded as a cause for the observed increased telomerase activity after irradiation, although direct evidence was lacking for our present data. Additionally, in a study investigating the effect of genetic factors on telomerase activity in PHA-stimulated PBLs, Kosciolek and Rowley [31] found a heritability of 0.814, indicating that genetic factors played a very critical role in determining the inducibility of telomerase activity, which may help maintain telomere structure and genetic stability. Therefore, one can speculate that dysregulation is present in the telomerase activation pathway in GC patients. In the present study, we hope to expect a similar effect of genetic factors on γ-radiation-induced telomerase activity in PBLs. However, unfortunately, the blood samples have not been collected from the relatives of subjects in our study, we could not provide experimental evidence of 'heritability'. Future study is needed to confirm our expectation.

In our study, stratified analysis was also performed by host characteristics. Our findings showed that individuals at least 60 years old had a significantly higher mean level of γ-radiation-induced telomerase activity than did those younger than 60 years among the GC cases (1.61 ± 1.08 vs. 1.39 ± 0.75; *p *= 0.060) and healthy controls (1.36 ± 0.76 vs. 1.07 ± 0.60; *p *< 0.001), suggesting a modulating effect of age on γ-radiation-induced telomerase activity. This result is consistent with a widely accepted concept that GC incidence increases with age. We hypothesize that, when exposed to the radiation, cells in older people will suffer from more severe DNA damage than those in younger people because DNA repair capacity is getting worse with age. Therefore, the level of telomerase activation as a response to DNA damage will be higher in older people than in younger people. To clearly address the molecular mechanism underlying the modulating effect of age on γ-radiation-induced telomerase activity, further investigations are urgently needed. Our data also showed that the increased GC risk associated with higher γ-radiation-induced telomerase activity was more evident in male person than in female person and also more evident in never-drinker than in ever drinker, indicating that males or never-drinkers may be more liable to suffer from telomerase activation upon mutagen challenge than females or ever-drinkers. If true, these observations in our study could point toward differences in the effects of some host characteristics on γ-radiation-induced telomerase activation. However, we could not rule out the possibility of chance findings because of the limited size in each subgroup. Further investigations are needed to confirm these findings.

Our study had two strengths. First, we used a case-control study design with a large sample size, which can significantly improve the statistical power. Second, we used the TRAP-ELISAplus, which includes an internal control to eliminate the effect of potential PCR inhibitors in the protein samples of cultured lymphocytes on the detection of telomerase activity, thereby assuring the reliability of the data. However, our study also had some limitations. Whether γ-radiation-induced telomerase activity detected in PBLs reflects that in gastric cells has yet to be determined. Also, in the present study, a case-control study was used to access the association of γ-radiation-induced telomerase activity and GC risk. Therefore, our study definitely has a limitation inherited from this study design, namely that we could not determine whether the observed increased telomerase activity was present prior to cancer development and therefore involved in cancer risk or whether it is a systemic biomarker of gastric cancer because the blood samples were drawn after diagnosis. However, our data showed that there were no significant associations between γ-radiation-induced telomerase activity and smoking status, drinking status and Hp infection in either GC cases or controls and that the level of γ-radiation-induced telomerase activity in cases was not significantly associated with the tumor stage and histological type, suggesting that ability of telomerase activation might be more likely to be determined by genetic but not environmental factors or disease itself and play a role at early stage of GC development. Further confirmation of the cause-and-effect relationship between γ-radiation-induced telomerase activity and GC development is warranted by using a prospective study design. In addition, the γ-radiation-induced telomerase activity in PBLs is only a biomarker for the assessment of telomerase inducibility but not clinical relevent event, which might attenuate the biological significance in carcinogenesis. Other biomarkers for risk evaluation in gastric cancer are still needed.

## Conclusion

Our data show for the first time that the level of γ-radiation-induced telomerase activity is significantly higher in GC cases than controls and the increased γ-radiation-induced telomerase activity is associated with increased GC risk. This study is an initial step to evaluate whether the higher inducibility of telomerase activity could be a risk factor for GC development and whether the induced telomerase activity could be used to assess GC risk. Further investigations are urgently needed to validate our findings and determine the causality and underlying mechanism of telomerase in GC development and progression.

## Competing interests

The authors declare that they have no competing interests.

## Authors' contributions

XH and QQ participated in the study design and coordination, collected the samples and drafted the manuscript. NG performed cell culture and telomerase activity measurement. JN performed data management. ZW performed statistical analysis. SS assisted with development of analytical plan. YY and GB participated in the design of the study and revised the manuscript critically. All authors read and approved the final manuscript.

## Pre-publication history

The pre-publication history for this paper can be accessed here:

http://www.biomedcentral.com/1471-2407/10/312/prepub
